# DRESS induced by amoxicillin-clavulanate in two pediatric patients confirmed by lymphocyte toxicity assay

**DOI:** 10.1186/s13223-021-00535-4

**Published:** 2021-04-05

**Authors:** Arun Dhir, Hasandeep Kular, Abdelbaset A. Elzagallaai, Bruce Carleton, Michael J. Rieder, Raymond Mak, Tiffany Wong

**Affiliations:** 1 Department of Medicine, University of British Columbia, Vancouver, BC Canada; 2 Division of Allergy and Immunology, Department of Medicine, University of British Columbia, Vancouver, BC Canada; 3 Department of Physiology and Pharmacology, Western University, London, ON Canada; 4 Department of Pediatrics, Western University, London, ON Canada; 5 Robarts Research Institute, Western University, London, ON Canada; 6 Division of Translational Therapeutics, Department of Pediatrics, University of British Columbia, Vancouver, BC Canada; 7 British Columbia Children’s Hospital Research Institute, University of British Columbia, Vancouver, BC Canada; 8 Pharmaceutical Outcomes Program, British Columbia Children’s Hospital, Vancouver, BC Canada; 9 CIHR-GSK Chair in Paediatric Clinical Pharmacology, Children’s Hospital of Western Ontario, London, ON Canada; 10 Division of Allergy and Immunology, Department of Pediatrics, University of British Columbia, Vancouver, BC Canada

**Keywords:** “Drug reaction with eosinophilia and systemic symptoms”, DRESS, Lymphocyte Toxicity Assay, Antibiotics, Amoxicillin-clavulanate

## Abstract

**Background:**

Drug reaction with eosinophilia and systemic symptoms (DRESS) is a rare but serious delayed hypersensitivity reaction that can be caused by antibiotic exposure. The reaction typically develops in 2 to 6 weeks. The pathophysiology is thought to involve toxic drug metabolites acting as a hapten, triggering a systemic response. The diagnosis is made clinically but can be confirmed using assays such as the lymphocyte toxicity assay (LTA), which correlates cell death upon exposure to drug metabolites with susceptibility to hypersensitivity reactions.

**Case presentations:**

Case 1 involves a previously healthy 11-month-old male with first exposure to amoxicillin-clavulanate, prescribed for seven days to treat a respiratory infection. The patient developed DRESS fourteen days after starting the drug and was successfully treated with corticosteroids. LTA testing confirmed patient susceptibility to hypersensitivity reactions with amoxicillin-clavulanate. Parental samples were also tested, showing both maternal and paternal susceptibility. Neither parent reported prior hypersensitivity reactions. Lifelong penicillin avoidance for the patient was advised along with the notation in medical records of penicillin allergy. The parents were advised to avoid penicillin class antibiotics and be monitored closely for DRESS if they are exposed.

Case 2 involves an 11-year-old female with atopic dermatitis with first exposure to amoxicillin-clavulanate, prescribed for ten days to treat a secondary bacterial skin infection. She developed DRESS eleven days after starting antibiotics and was successfully treated with corticosteroids. LTA testing confirmed patient susceptibility to hypersensitivity reactions with amoxicillin-clavulanate. Maternal samples were also tested and showed sensitivity. The mother reported no prior hypersensitivity reactions. Lifelong penicillin avoidance for the patient was advised along with the notation in medical records of penicillin allergy.

**Conclusions:**

Amoxicillin-clavulanate is a commonly used antibiotic and the cases we have described suggest that it should be recognized as a potential cause of DRESS in pediatric patients. Furthermore, these cases contribute to current literature supporting that there may be a shorter latent period in DRESS induced by antibiotics. We have also shown that the LTA can be a helpful tool to confirm DRESS reactions, and that testing may have potential implications for family members.

## Background

Drug reaction with eosinophilia and systemic symptoms (DRESS) is a rare but potentially fatal delayed hypersensitivity reaction. It is hypothesized that the reaction involves a combination of the activation of drug-specific T-lymphocytes, latent viral reactivation, accumulation of reactive drug metabolites, as well as genetic predisposition [1]. The toxic metabolite acts as a hapten, initiating an immune response. DRESS is classically associated with anticonvulsant agents, but 15–37% of DRESS cases may be due to antibiotics, [2] with one with study reporting up to 74% (39% to vancomycin, 23% to beta-lactams) [3]. The reaction typically has a latency period of 2 to 6 weeks [2, 4]. Symptoms include fever, diffuse rash, lymphadenopathy, hematologic abnormalities (eosinophilia, atypical lymphocytosis), and ultimately internal organ involvement [2]. This diagnosis is made clinically, often supported by tools such as the European Registry of Severe Cutaneous Adverse Reaction (RegiSCAR) validation scoring criteria [5].

Additional tools that may be used to confirm a diagnosis of DRESS include patch testing and in-vitro assays. The lymphocyte toxicity assay (LTA) is one such assay based upon based upon the premise that DRESS may be triggered by accumulation of toxic metabolites [6]. The patient’s lymphocytes, isolated from peripheral blood samples and acting as a surrogate for target tissue cells, are incubated with the suspected drug in presence of phenobarbital-induced mammalian hepatic microsomes as a source of cytochrome P450 monooxygenase activity. The degrees of cell death in samples isolated from patients and from healthy volunteers are then quantified and compared. Enhancement of cell death is hypothesized to correlate with the patient’s susceptibility to developing hypersensitivity reactions to the agent being tested.

Here, we present two cases of pediatric patients with DRESS induced by amoxicillin-clavulanate and the results of their LTA testing.

## Case presentations

**Fig. 1:**
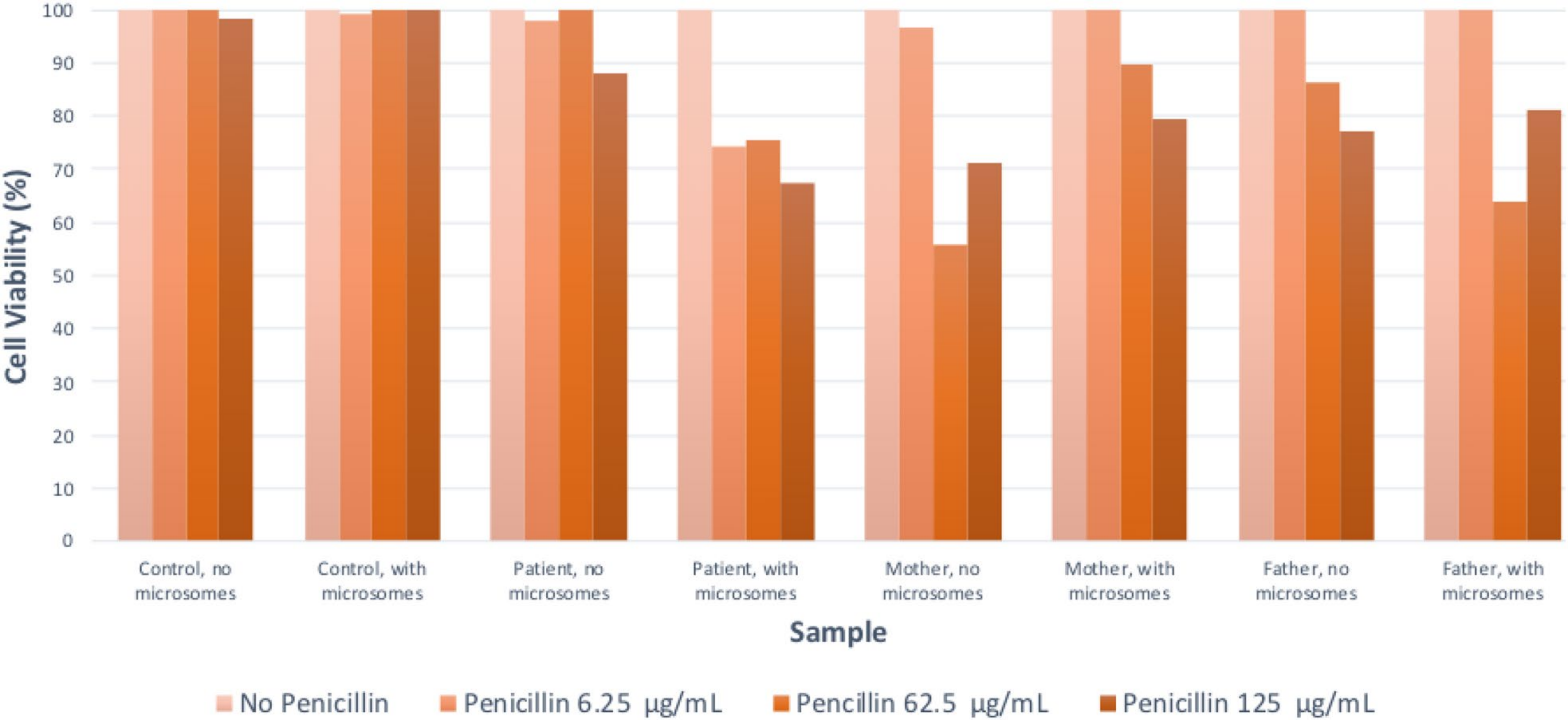
Case 1 lymphocyte toxicity assay

Case 1 involves a previously healthy 11-month-old male with first exposure to amoxicillin-clavulanate, prescribed for seven days to treat a respiratory infection. Fourteen days after starting antibiotics, he presented with fevers, lethargy, and a widespread generalized erythematous maculopapular rash. Laboratory investigations showed reactive lymphocytes, peripheral eosinophilia, and hepatitis. Testing for ANA, hepatitis A and B, EBV, CMV, HHV6, mycoplasma, chlamydia, and blood cultures were negative. A RegiSCAR Diagnosis Score of 6 confirmed definite DRESS (Table 1). The patient received systemic steroids, resulting in normalization of lab work and improvement of symptoms. LTA testing showed a concentration-dependent decline in viability in the patient’s white blood cells compared to controls after incubation with penicillin and its metabolites (Fig. 1). Lifelong penicillin avoidance was advised along with the notation in medical records of penicillin allergy. Parental samples were tested, showing a concentration-dependent decline in cell viability when exposed to penicillin and its metabolites. Neither parent reported prior adverse reactions to antibiotics. They were counselled that the implications of a positive LTA without a prior reaction are unknown. They were also advised that alternatives to penicillin class antibiotics should be used to treat infections, and that if exposed to such antibiotics, they should be monitored closely for symptoms of DRESS.

**Table 1:** RegiSCAR Scoring System for Classifying DRESS Cases, adapted from Cho et al. [5] applied to patients described in Case 1 and Case 2

Items	Score			Comments	Case 1 score	Case 2 score
	− 1	0	1			
Fever ≧ 38.5 °C	N/U	Y			0	0
Enlarged lymph nodes		N/U	Y	> 1 cm and ≧ 2 different areas	0	0
Eosinophilia ≧ 0.7 × 10^9^/L or ≧ 10% if WBC < 4.0 × 10^9^/L		N/U	Y	Score 2, when ≧ 1.5 × 10^9^/L or ≧ 20% if WBC < 4.0 × 10^9^/L	1	1
Atypical lymphocytosis		N/U	Y		1	0
Skin rash				Rash suggesting DRESS: ≧ 2 symptoms: purpuric lesions (other than legs), infiltration, facial edema, psoriasiform desquamation		
Extent > 50% of BSA		N/U	Y		1	1
Rash suggesting DRESS	N	U	Y		1	1
Skin biopsy suggesting DRESS	N	Y/U			0	0
Organ involvement		N	Y	Score 1 for each organ involvement, maximal score: 2	1	1
Rash resolution ≧ 15 days	N/U	Y			0	0
Excluding other causes		N/U	Y	Score 1 if 3 tests of the following tests were performed and all were negative: HAV, HBV, HCV, Mycoplasma, Chlamydia, ANA, blood culture	1	1
Total score					6	5

The diagnosis of DRESS syndrome is then made based on the total score: < 2 points: no case; 2–3 points: possible case; 4–5 points: probable case; > 5 points: definite case

ANA: anti-nuclear antibody; BSA: body surface area; HAV: hepatitis A virus; HBV: hepatitis B virus; HCV: hepatitis C virus; N: no; U: unknown; WBC: white blood cell; Y: yes

**Fig. 2:**
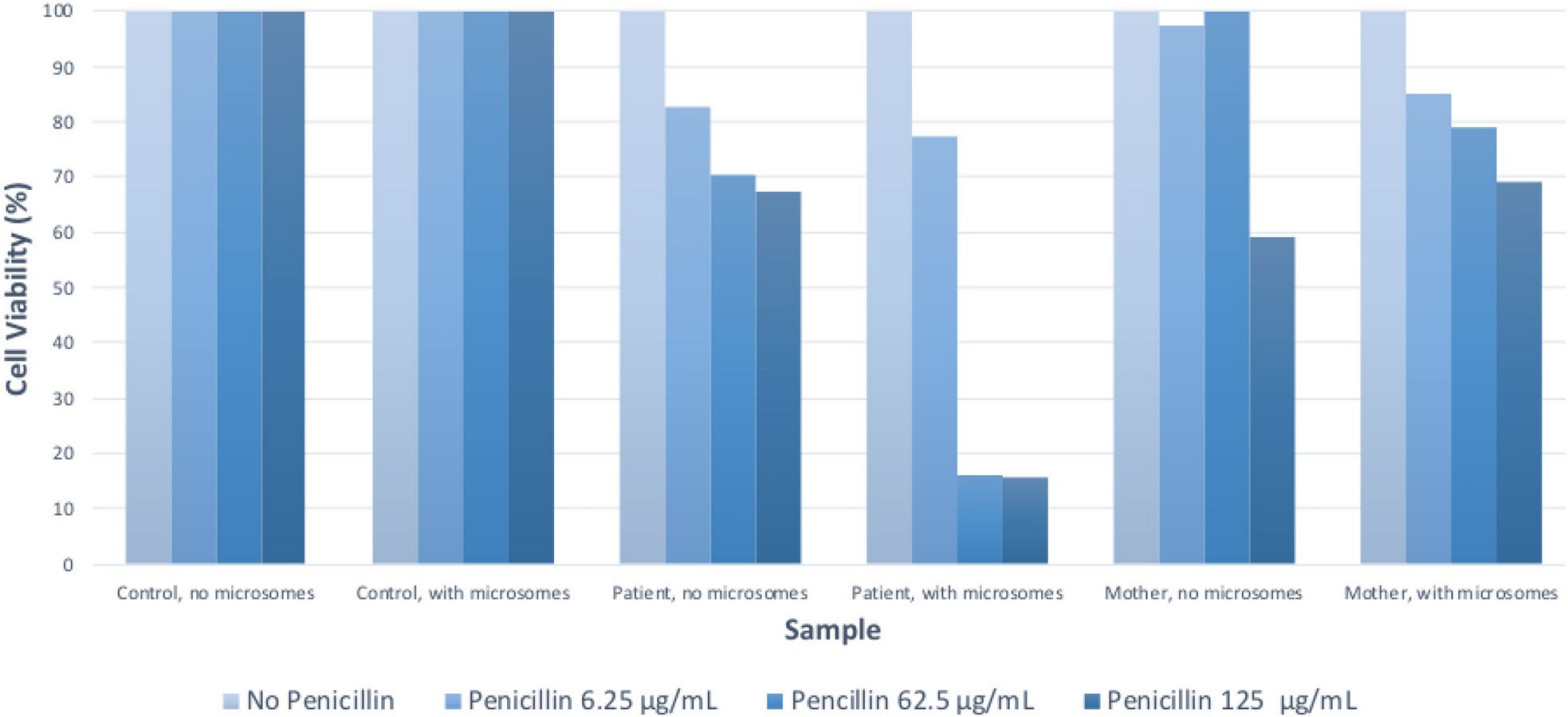
Case 2 lymphocyte toxicity assay

Case 2 involves an 11-year-old female with atopic dermatitis with a first exposure to amoxicillin-clavulanate, prescribed for ten days to treat a secondary bacterial skin infection. She developed fevers and decreased appetite eleven days after starting antibiotics. This progressed to diffuse erythrodermic, maculopapular eruption, superficial desquamation, facial angioedema, peripheral eosinophilia, hepatitis, and lymphadenopathy. The patient’s ANA and blood cultures were negative. Her RegiSCAR-Group Diagnosis Score was 5, suggesting probable DRESS (Table 1). LTA testing showed a substantial decline in viability of the patient’s white blood cells compared to controls when exposed to penicillin and its metabolites (Fig. 2). The patient received systemic corticosteroids, resulting in normalization of lab work and improvement of symptoms. Lifelong penicillin avoidance was advised along with the notation in medical records of penicillin allergy. Maternal samples also showed concentration-dependent decreased cell viability. The patient’s mother had no prior adverse reactions to antibiotics.

Case 1 lymphocyte toxicity assay results show a concentration-dependent decline in patient and parent lymphocyte viability when incubated with penicillin and activated mammalian-derived microsomes. Compared to control cells, the patient and parent cells demonstrated decreased viability when incubated with penicillin, and showed further decline when incubated with penicillin metabolites generated by the activated microsomes.

Case 2 lymphocyte toxicity assay results show a concentration-dependent decline in patient and parent lymphocyte viability when incubated with penicillin and activated microsomes. Compared to control cells, the patient and parent cells demonstrated decreased viability when incubated with penicillin, and showed further decline when incubated with penicillin metabolites generated by the activated microsomes.

## Discussion

Few cases of DRESS associated with amoxicillin-clavulanate have been published, particularly in children. One case report described a 12-year-old male who received empiric amoxicillin-clavulanate for a fever and cough and subsequently developed DRESS within a ten day period [7]. Similarly, our patients developed symptoms within two weeks as opposed to the typically reported 2 to 6 week period, supporting the possibility of a shorter latency period for DRESS induced by antibiotics in children [2, 4].

Research on the sensitivity and specificity of the LTA is limited, partly due to the test being limited to well-equipped research centers and validated only for few classes of drugs. In their retrospective study, Elzagallaii et al*.* found that amongst 13 patients with re-exposure events to beta-lactam antibiotics, the LTA showed a sensitivity of 40% and a specificity of 100% [8]. The cases we have described further suggest that the LTA may be a useful tool in the evaluation of DRESS reactions.

The use of LTA to risk stratify relatives of patients who have had DRESS has not been previously reported. With regards to Case 1, both parents had abnormal LTA results, suggesting a genetic contribution to the patient’s sensitivity. An association between human leukocyte antigen haplotypes and susceptibility to DRESS is well established for allopurinol, carbamazepine, abacavir and other medications, [2] although no such strong association has yet been discovered with penicillin-class antibiotics. The LTA test supports the hapten hypothesis, i.e. that reactive metabolites contribute to the development of DRESS via hapten formation and assessment of white blood cell toxicity can serve to identify phenotypic vulnerability in patient cells [8].

## Conclusions

Amoxicillin-clavulanate is a commonly used antibiotic and the cases we have described suggest that it should be recognized as a potential cause of DRESS in pediatric patients. Furthermore, these cases contribute current literature supporting that there may be a shorter latent period in DRESS induced by antibiotics. We have also shown that the LTA can be a helpful tool to confirm DRESS reactions, and that testing may have potential implications for family members.

## Data Availability

The datasets used and analysed during the current study are available from the corresponding author on reasonable request.

## Abbreviations

DRESS: Drug reaction with eosinophilia and systemic symptoms
LTA: Lymphocyte toxicity assay
